# Prevention, treatment, and risk factors of deep vein thrombosis in critically ill patients in Zhejiang province, China: a multicenter, prospective, observational study

**DOI:** 10.1080/07853890.2021.2005822

**Published:** 2021-11-19

**Authors:** Li Li, Jia Zhou, Liquan Huang, Junhai Zhen, Lina Yao, Lingen Xu, Weimin Zhang, Gensheng Zhang, Qijiang Chen, Bihuan Cheng, Shijin Gong, Guolong Cai, Ronglin Jiang, Jing Yan, (Zhejiang Provincial Critical Care Clinical Research Group)

**Affiliations:** a Department of Critical Care Medicine, Zhejiang Hospital, Hangzhou, Zhejiang, China; b Department of Critical Care Medicine, Zhejiang Provincial Hospital of Chinese Medicine, Hangzhou, Zhejiang, China; c Department of Critical Care Medicine, Ningbo Yinzhou People’s Hospital, Yinzhou, Zhejiang, China; d Department of Critical Care Medicine, Xinchang Hospital of Traditional Chinese Medicine, Xinchang, Zhejiang, China; e Department of Critical Care Medicine, Dongyang People's Hospital, Dongyang, Zhejiang, China; f Department of Critical Care Medicine, The Second Affiliated Hospital Zhejiang University School of Medicine, Hangzhou, Zhejiang, China; g Department of Critical Care Medicine, Ninghai First Hospital, Ninghai, Zhejiang, China; h Department of Critical Care Medicine, The 2^nd^ School of Medicine, Wenzhou Medical University, Wenzhou, Zhejiang, China

**Keywords:** Deep vein thrombosis, prevention, treatment, risk factors, intensive care unit, China

## Abstract

**Purpose:**

The aim of this study is to investigate the prevention and treatment patterns of deep vein thrombosis (DVT) in critically ill patients and to explore the risk factors for DVT in people from Zhejiang Province, China.

**Materials and methods:**

This study prospectively enrolled patients admitted in intensive care units (ICUs) of 54 hospitals from 09/16/2019 to 01/16/2020. The risk of developing DVT and subsequent prophylaxis was evaluated. The primary outcome was DVT occurrence during ICU hospitalisation. Univariate and multivariate logistic regression were performed to determine the risk factors for DVT.

**Results:**

A total of 940 patients were included in the study. Among 847 patients who received prophylaxis, 635 (75.0%) patients received physical prophylaxis and 199 (23.5%) patients received drug prophylaxis. Fifty-eight (6.2%) patients were diagnosed with DVT after admission to the ICU, and 36 patients were treated with anticoagulants (all patients received low molecular weight heparin [LMWH]). D-dimer levels (OR = 1.256, 95% CI: 1.132–1.990), basic prophylaxis (OR = 0.092, 95% CI: 0.016–0.536), and physical prophylaxis (OR = 0.159, 95% CI: 0.038–0.674) were independently associated with DVT in ICU patients. The short-term survival was similar between DVT and non-DVT patients.

**Conclusions:**

DVT prophylaxis is widely performed in ICU patients. Prophylaxis is an independent protective factor for DVT occurrence. The most common treatment of DVT patients is LMWH, although it might increase the rate of bleeding.

**Key messages**
- This is the only multicenter and prospective study of DVT in ICUs in China.
- d-dimer levels were independently associated with DVT in ICU patients.
- Prophylaxis was an independent protective factor for DVT occurrence in ICU.

## Introduction

Deep vein thrombosis (DVT) is caused by a blood clot obstructing blood flow in the deep venous system, most commonly occurring in the lower limbs [1]. It can be provoked by factors, such as recent surgery or trauma, hospitalisation with prolonged bed rest, or the use of oral contraceptives [1]. An unprovoked DVT may be idiopathic or inherited or from acquired hypercoagulable states, such as cancer and pregnancy [1]. The estimated annual worldwide incidence of venous thromboembolism (VTE) is 10 million [1–3]. DVT is observed in about 0.51% of hospitalised patients [4], 0.8%–8% of patients with cancer [5–7], 0.8%–9.6% of patients with surgery [8–10], and 5%–31% of intensive care unit (ICU) patients [11,12], and the incidence increases with age [1–3]. A study based on a large healthcare database in China revealed an annual incidence of DVT of 30.0 per 100,000 populations [13]. Pulmonary embolism, post-thrombotic syndrome, phlegmasia cerulea dolens, phlegmasia alba dolens, and paradoxical embolism leading to cryptogenic embolic stroke are possible complications of DVT [14–17]. In addition, DVT is associated with an increased risk of mortality [18–20].

The risk factors for DVT include transient or persistent factors that may additively or synergistically increase thrombotic risk by causing vascular wall damage or dysfunction, stasis, or blood hypercoagulability (Virchow’s triad) [2,3]. Patients in the ICU carry multiple risk factors for DVT, including long-term immobilisation, sedative and analgesic use, deep vein catheterisation, tracheotomy, surgery or invasive procedures, and possibly cancer, among others. All these factors will increase the risk of DVT in a population of patients already at high risk of morbidity and mortality [21–23]. Therefore, the prevention of DVT is crucial for ICU patients. Nevertheless, most studies about the epidemiology, prevention, and treatment of DVT in ICUs are from Western countries, and data on developing countries, such as China, are lacking. Among 80 ICU patients over 9 months at a hospital in Hong Kong, the incidence of DVT in the ICU was 15 patients with an incidence rate of 18.8% [24].

The prevention of VTE in hospitalised patients is a clinical challenge because it requires balancing the risk of developing DVT and DVT-related complications and the risk of prophylaxis-related complications, mainly bleeding. Presently, the recommendations for critically ill patients indicate that pharmacological prophylaxis (using unfractionated heparin [UFH] or low molecular weight heparin [LMWH]) should be preferred over mechanical prophylaxis. However, if the patient is bleeding or at high risk of bleeding, mechanical prophylaxis should be considered until bleeding stops or bleeding risk decreases, then switching to pharmacological prophylaxis should be considered [25,26].

Therefore, the objective of this multicenter study was to investigate the prevention and treatment patterns of DVT in critically ill patients and to explore the risk factors for DVT. This study could provide evidence for improving the management of DVT in critically ill patients.

## Patients and methods

### Study design and patients

This study prospectively enrolled patients admitted in the ICUs of 54 hospitals in Zhejiang Province, China from September 16, 2019 to January 16, 2020. The characteristics of the hospitals are presented in Supplementary Table S1. All ICU patients >18 years of age were included in this study. The exclusion criteria were (1) patients diagnosed with DVT or pulmonary embolism before admission to the ICU or (2) with an expected ICU stay of <48 h.

This study involving human participants was in accordance with the ethical standards of the institutional and national research committee and the 1964 Helsinki Declaration and its later amendments or comparable ethical standards. The study was approved by the ethics committee of all participating ICUs (2019-24 K) and registered (ChiCTR1900024956). The written informed consent was obtained from the legal representative of patients.

### DVT risk assessment

The DVT risk assessment scales used in this study were the Caprini risk score scale [27], Padua risk score scale [28], and Wells DVT risk assessment scale [29]. The first assessment was performed within 48 h of admission and reassessment was performed when the patients’ condition changed, or regularly. A change in the condition was defined as (1) blood pressure < 90/60 mmHg or dropped by >30%, (2) PO_2_ <60 mmHg, or (3) requirement of an invasive operation or emergency surgery. The frequency of routine reassessment included once a month, once a week, twice a week, and daily.

### DVT prophylaxis

The specific preventive measures for VTE were conducted by following the 2018 Chinese Thoracic Society “Guidelines for the Diagnosis, Treatment, and Prevention of Pulmonary Thromboembolism” and the 2020 “Chinese Expert Consensus on Mechanical Prevention of Venous Thromboembolism.”

The basic prevention measures included rehabilitation exercises for lower limbs and avoidance of dehydration. The specific measures included controlling the blood lipids and blood glucose, raising the affected limb, and early functional training. Drug prophylaxis for patients at a high risk of VTE and a low risk of haemorrhage included UFH, LMWH, fondaparinux, new oral anticoagulants, and vitamin K antagonists. In patients who received long-term drug prophylaxis, the effectiveness of prevention of VTE and the potential risk of bleeding were dynamically evaluated. Patients at a high risk of VTE but with active haemorrhage or at risk of haemorrhage were given physical prevention, including intermittent pneumatic compression (IPC), graduated compression stockings (GCS), and venous foot pumps (VFPs). IPC was of two types: knee-length and leg-length and was used by the orders of the ICU doctors and the conditions of the hospitals. It was used every day for more than 18 h, during which the patient’s condition and the equipment were evaluated. GCS included three types: knee-length, leg-length, and continuous waist. The size of the GCS was selected according to the smallest circumference of the patient’s ankle, the largest circumference of the calf, and the circumference of the central part of the groyne 5 cm down. GCS was generally worn by the patients for the whole day, and the conditions of these patients and the GCS were evaluated daily during hospitalisation. The timing and frequency of VFPs were the same as for IPC.

### DVT diagnosis and treatment

The diagnostic methods and criteria of DVT were according to the 2017 “Guidelines for the Diagnosis and Treatment of Deep Vein Thrombosis (Third Edition)” by the Chinese Society of Vascular Surgery of the Chinese Medical Association.

The treatment of DVT was conducted according to the 2017 “Guidelines for the Diagnosis and Treatment of Deep Vein Thrombosis (Third Edition)” by the Chinese Society of Vascular Surgery of the Chinese Medical Association and the 2018 Chinese Thoracic Society “Guidelines for the Diagnosis, Treatment, and Prevention of Pulmonary Thromboembolism.” The patients were treated with one drug among UFH, LMWH, and vitamin K antagonists. Moreover, the therapy was changed according to the condition of the patient. The initial dose of UFH was 80–100 U/kg/h intravenously, and then 10–20 U/kg/h intravenously. Afterward, it was adjusted according to the activated partial thromboplastin activity every 4–6 h to extend it to 1.5–2.5 times the normal control value. LMWH was administered according to the bodyweight at a dose of 100 U/kg once every 12 h, subcutaneously. The initial dose of vitamin K antagonists, such as warfarin, was 3.0–5.0 mg/d, starting with 2.5–3.0 mg for patients >75 years old and at high haemorrhage risk. Thrombolysis therapy included streptokinase, urokinase, and recombinant human tissue plasminogen activator (rt-PA), each used alone. The loading dose of streptokinase was 250,000 U intravenously for 30 min, followed by a maintenance intravenous infusion of 100,000 U/h for 12–24 h. The loading dose of urokinase was 4400 U/kg intravenously for 10 min, followed by 2200 U/kg/h continuous intravenous drip for 12 h. The dose of rt-PA was 50 mg, infused continuously for 2 h.

### Data collection and follow-up

The baseline characteristics of the patients were collected, including age, sex, height, weight, vital signs, vasoactive drug use, deep vein indwelling, APACHE II score, laboratory tests, and vascular ultrasound Doppler examination results. The laboratory tests were performed within 24 h of admission. Patients were followed up during the hospital stay. The primary outcome was DVT occurrence during ICU hospitalisation. The secondary outcomes were the 28- and 60-day survival rates, time of ICU stay, total hospital stay, pulmonary embolism, haemorrhage events, and coagulopathy within 60 days.

### Sample size

The incidence of DVT in ICU in China is 11.9% [30]. Using a power of 80% and a significance level of .05, and a 15% dropout rate, 792 patients would need to be enrolled in the current study.

### Statistical analysis

SPSS 26.0 (IBM, Armonk, NY, USA) was used for the statistical analysis. The continuous data with a normal distribution (according to the Kolmogorov–Smirnov test) were presented as mean ± standard deviation and tested using Student’s *t*-test; otherwise, they were presented as median (range) and were analysed using the Mann–Whitney *U*-test. The categorical variables were presented as *n* (%) and analysed using the chi-square test. Based on propensity score matching (PSM), a logistic regression model was used to match the enrolled patients from the DVT and the non-DVT groups. Reasons for the admission of patients to ICU and the admission department before ICU were used as a matching variable to estimate the PS value of each patient. Every patient in the DVT group was matched to a patient in the non-DVT group with a PS difference not exceeding 0.03 (matching tolerance). If there were multiple individuals within the matching tolerance of 0.05, a random sampling method was adopted to select one for matching. After balancing the two basic characteristics, univariable logistic regression was used to analyse the risk factors for DVT. A multivariable logistic regression model was used to determine the independent risk factors for DVT events. The Cronbach's alpha was used to assess the scale for validity and Kaiser-Meyer-Olkin was used to assess the scale for reliability. For all analyses, two-sided *P*-values <.05 were considered statistically different.

## Results

### Characteristics of patients

**Figure 1.**
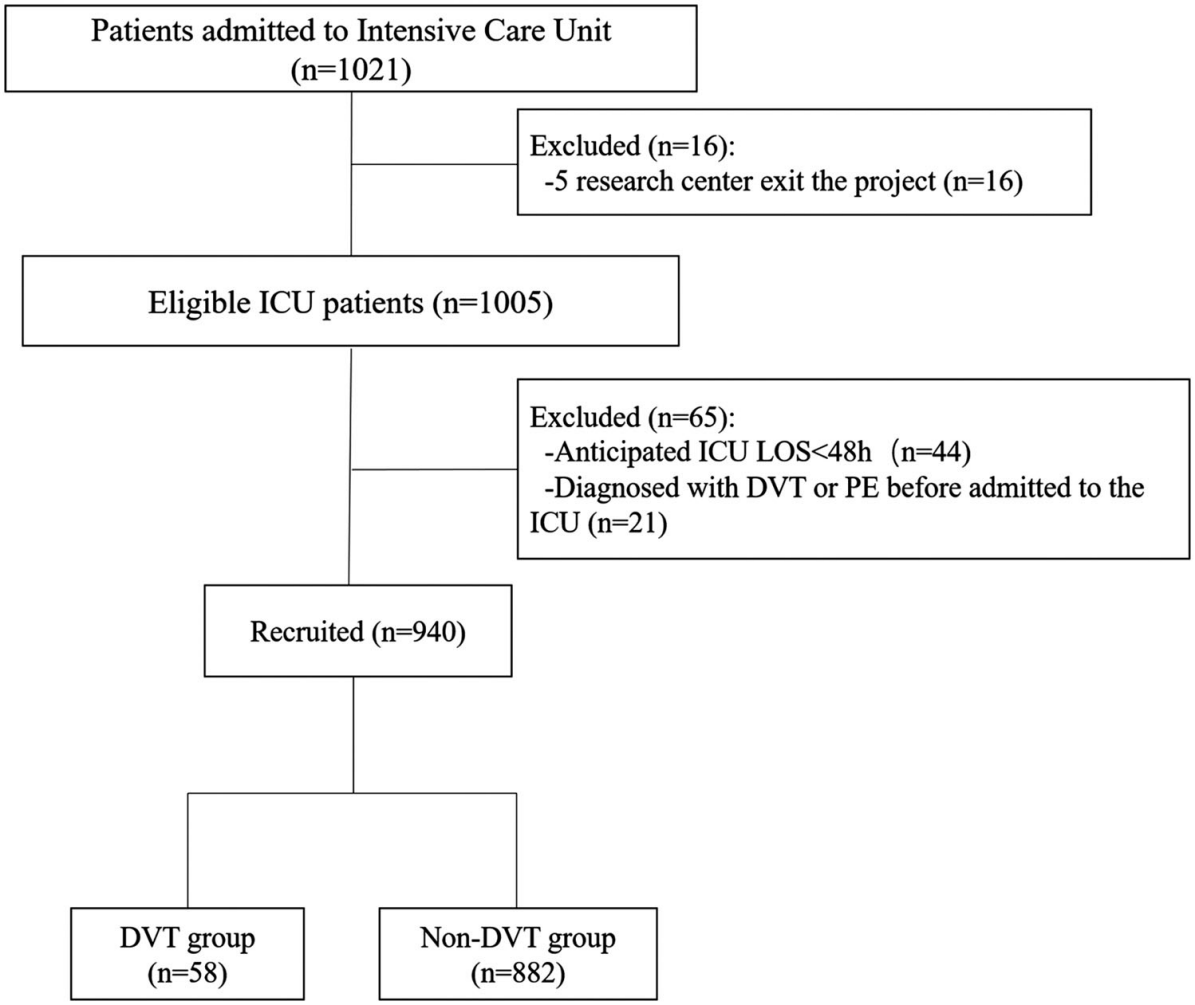
Patient flowchart.

A total of 1021 patients who met the inclusion criteria and were admitted in 54 ICUs from September 16, 2019 to January 16, 2020, were first enrolled in the study. Then, 65 patients were excluded based on the exclusion criteria. During follow-up, 16 more patients from five centres were excluded because these centres withdrew from the study. Finally, 940 patients were included in the analyses (Figure 1). The characteristics of the patients are shown in Table 1.

**Table 1.** Characteristics of the patients.

Characteristics	Total (*n* = 940)	Before matching			After matching		
		DVT group (*n* = 58)	Non-DVT group (*n* = 882)	*P*	DVT group (*n* = 58)	Non-DVT group (*n* = 882)	*P*
Age, years, median (range)	69 (18–99)	66 (32–91)	69 (18–90)	.698	67 (32–91)	66 (21–99)	0.784
Male, *n* (%)	636 (67.7)	39 (67.2)	597 (67.9)	.944	39 (67.2%)	33 (56.9%)	0.251
BMI, kg/m^2^, mean ± SD	22.5 (9.9–34.6)	22.7 (16.7–32.0)	22.5 (9.9–35.1)	.358	22.8 (16.8–32.0)	23.2 (16.6–42.7)	0.388
APACHE II, median (range)	18.0 (4.0–51.0)	17.0 (3.0–34.0)	18.0 (1.0–51.0)	.800	17.0 (3.0–34.0)	16.0 (3.0–42.0)	0.538
Underlying disease, *n* (%)							
Chronic lung disease	178 (18.9)	29 (50)	149 (16.9)	.007	2 (3.4)	6 (10.3)	0.272
Hypertension	455 (48.4)	26 (44.8)	429 (48.6)	.574	26 (44.8)	27 (46.6)	0.852
Diabetes	166 (17.7)	11 (18.9)	155 (17.6)	.788	11 (19.0)	10 (17.2)	0.809
Heart disease	223 (23.7)	10 (17.2)	213 (24.1)	.231	10 (17.2)	16 (27.6)	0.182
Renal disease	69 (7.3)	2 (3.4)	67 (7.6)	.361	2 (3.4)	7 (12.1)	0.165
Gastrointestinal disease	34 (3.6)	0	34 (3.9)	.246	0	3 (5.2)	0.242
Peripheral vascular disease	21 (2.2)	0	21 (2.4)	.465	0	2 (3.4)	0.476
Long–term catheter indwelling	67 (7.1)	5 (8.6)	62 (7.0)	.847	5 (8.6)	4 (6.9)	>0.999
Cerebrovascular diseases	153 (16.3)	4 (6.9)	149 (16.9)	.046	4 (6.9)	12 (20.7)	0.031
Tumour history	86 (9.1)	4 (6.9)	82 (9.3)	.539	4 (6.9)	8 (13.8)	0.223
History of surgery	181 (19.3)	9 (15.5)	172 (19.5)	.456	9 (15.5)	21 (36.2)	0.011
Comorbidity, *n* (%)							
Sepsis	108 (11.5)	5 (8.6)	103 (11.7)	.479	5 (8.6)	6 (10.3)	0.751
Sepsis shock	82 (8.7)	2 (3.4)	80 (9.1)	.142	2 (3.4)	3 (5.2)	>0.999
Acute kidney injury	138	6 (10.3)	132 (15.0)	.335	6 (10.3)	4 (6.9)	0.508
Admission department before ICU, *n* (%)							
Emergency room	468 (49.8)	32 (55.2)	436 (49.4)	.397	32 (55.2)	39 (67.2)	0.182
Other general ward	257 (27.3)	6 (10.3)	251 (28.5)	.003	6 (10.3)	7 (12.1)	0.769
Operating room	171 (18.2)	16 (27.9)	155 (17.6)	.056	16 (27.6)	6 (10.3)	0.018
Others	44 (4.6)	4 (6.9)	40 (4.5)	.614	4 (6.9)	6 (10.3)	0.508
Reasons for admission to ICU, *n* (%)							
Internal medicine disease	569 (60.5)	22 (37.9)	547 (62.0)	<.001	22 (37.9)	26 (44.8)	0.451
Surgical disease	257 (27.3)	32 (55.1)	225 (25.5)	<.001	32 (55.2)	32 (55.2)	>0.999
Trauma	76 (8.1)	4 (6.8)	72 (8.2)	.925	4 (6.9)	0	0.127
Others (poisoning etc.)	577 (61.4)	22 (37.9)	555 (62.9)	<.001	22 (37.9)	26 (22.8)	0.451
Other treatments, *n* (%)							
Deep vein catheterisation	529 (56.3)	38 (65.5)	491 (55.7)	.143	38 (65.5)	35 (60.3)	0.564
Mechanical ventilation	624 (66.4)	44 (75.9)	580 (65.7)	.115	44 (75.9)	35 (60.3)	0.073
Analgesic and sedative drugs	562 (59.8)	45 (77.6)	517 (58.2)	.004	45 (77.6)	31 (53.4)	0.006
Muscle relaxants	25 (26.4)	4 (6.9)	21 (2.3)	.099	4 (6.9)	0	0.127
CRRT	84 (8.9)	3 (5.2)	81 (9.2)	.300	3 (5.2)	4 (6.9)	>0.999
IABP	4 (0.4)	0	4 (0.4)	>.999	0	0	–
ECMO	7 (0.7)	0	7 (0.8)	>.999	0	0	–
Laboratory parameters, median (range)							
WBC, ×10^9^/L	11.2 (2.0–107.0)	17.0 (1.6–37.3)	11.3 (0.2–107.0)	.364	11.0 (1.6–37.3)	11.1 (3.0–70.4)	0.829
Lactate, mmol/L	1.9 (0–17.0)	2.0 (0.4–17.0)	1.9 (0.1–13.0)	.937	2.0 (0.4–17.0)	1.8 (0.5–15.0)	0.771
CRP, mg/L	45.0 (1.0–617.0)	67.9 (0.1–320.0)	44.0 (0.1–617.0)	.144	67.9 (0.1–320.0)	50.0 (1.6–277.7)	0.638
APTT, s	33.3 (12.9–47.1)	36.4 (20.8–47.0)	32.7 (12.0–44.3)	.004	36.3 (20.8–56.0)	36.0 (17.3–29.4)	0.821
INR	1.1 (0.9–5.1)	1.2 (0.9–5.1)	1.1 (0.8–3.7)	.096	1.2 (0.9–4.1)	1.1 (0.9–4.0)	0.280
D–dimer, mg/dl	2.4 (0.1–25.0)	12.0 (1.4–25.0)	2.2 (0.04–15.0)	<.001	12.0 (1.4–16.8)	2.7 (0.1–5.5)	<0.001
DVT risk assessment, *n* (%)							
Padua-low risk	52 (5.5)	1 (1.8)	51 (5.8)	.311	1 (1.7)	3 (5.2)	0.611
Padua-high risk	208 (22.1)	11 (19.0)	197 (22.3)	.549	11 (19.0)	12 (20.7)	0.816
Caprini-very low risk	2 (0.2)	0	2 (0.2)	>.999	0	0	–
Caprini-low risk	26 (2.8)	1 (1.7)	25 (2.8)	.931	1 (1.7)	0	>0.999
Caprini- medium risk	68 (7.2)	4 (6.9)	64 (7.3)	>.999	4 (6.9)	3 (5.2)	>0.999
Caprini-high risk	508 (54.0)	40 (69.0)	468 (53.1)	.019	40 (69.0)	34 (58.6)	0.246
Wells-low risk	1 (0.1)	0	1 (0.1)	>.999	0	0	–
Wells-median risk	74 (7.9)	1 (1.7)	73 (8.3)	.123	1 (1.7)	6 (10.3)	0.119
Wells-high risk	1 (0.1)	0	1 (0.1)	>.999	0	0	–
DVT score evaluation frequency, *n* (%)							
Within 24 h after admission	582 (61.9)	29 (50.0)	553 (62.7)	.054	29 (50.0)	36 (62.1)	0.190
Within 48 h after admission	868 (92.3)	53 (91.4)	815 (92.4)	.977	53 (91.4)	58 (100)	0.067
Changes in condition	43 (4.6)	23 (39.7)	20 (2.3)	<.001	23 (39.7)	1 (1.7)	<0.001
Routine reassessment	176 (18.7)	33 (56.9)	143 (16.2)	<.001	33 (56.9)	5 (8.6)	<0.001
DVT prophylaxis, *n* (%)							
Physiotherapy prophylaxis	798 (84.9)	33 (56.9%	765 (86.7)	<.001	33 (56.9)	55 (94.8)	<0.001
Compression therapy	660 (70.2)	25 (43.1)	635 (72.0)	<.001	25 (43.1)	50 (86.0%)	<0.001
Medication prophylaxis	207 (22.0)	8 (13.8)	199 (22.6)	.118	8 (13.8)	7 (12.1)	0.782

DVT: deep vein thrombosis; BMI: body mass index; APACHE: Acute Physiology and Chronic Health Evaluation; ICU: intensive care unit; CRRT: continuous renal replacement therapy; IABP: intra-aortic balloon pump; ECMO: extracorporeal membrane oxygenation; WBC: white blood cells; CRP: C-reactive protein; APTT: activated partial thromboplastin time; INR: international normalised ratio.

### DVT prophylaxis

**Figure 2.**
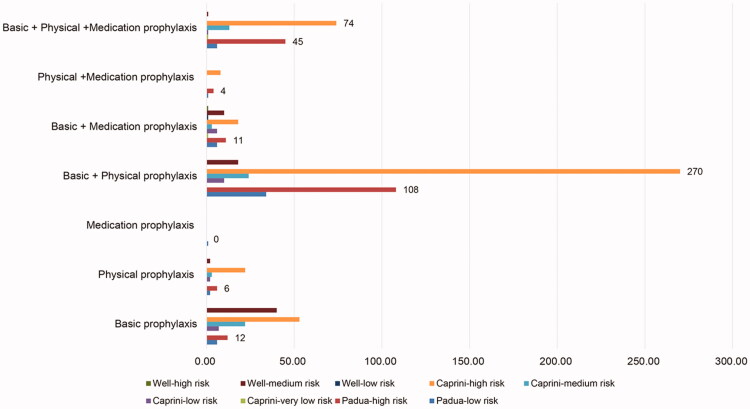
Prophylaxis of different deep vein thrombosis (DVT) risk levels.

Among the included patients, DVT prophylaxis was not conducted in 93 patients. The remaining 847 patients were given different prophylactic measures for thrombosis. Among them, 765 (90.3%) patients received basic prophylactic methods for thrombosis prevention. The physical methods for the prevention of DVT were used in 635 (75.0%) patients, of which, 523 (82.4%) patients used IPC devices, 15 (2.4%) used GCS devices, 60 (9.4%) used VFR, and the remaining 37 (5.8%) used multiple devices. Among the 847 patients, 199 (23.5%) patients were treated with drug prophylaxis. Among them, 149 (74.9%) patients received LMWH, 6 (3.0%) received UFH, 5 (2.5%) received warfarin, 35 (17.6%) received antiplatelet prophylaxis, and the remaining 4 (2.0%) took other drugs for the prevention of DVT. The prevention patterns of DVT are shown in Figure 2.

### DVT treatment

In total, 58 patients were diagnosed with DVT after entering the ICU. The thrombus was located in the popliteal vein (*n* = 5), femoral vein (*n* = 8), internal jugular vein (*n* = 2), posterior tibial vein (*n* = 2), lower extremity intermuscular vein (*n* = 37), and mixed location (*n* = 4). Once diagnosed with DVT, all patients were treated immediately using general treatment, including bed rest, and raising the affected limb. Thirty-six patients received anticoagulant therapy, of which 36 patients received LMWH, and 2 received warfarin. The ultrasound examination of six patients on the day of transfer indicated that the thrombus was completely recanalized. In one patient, who received only antiplatelet therapy and was transferred out of the ICU, an ultrasound scanning indicated that the thrombus had disappeared. Two patients were treated with anticoagulants combined with antiplatelet therapy, and their re-examination on the day of transfer from the ICU revealed that the thrombus had disappeared in one patient. Two patients underwent interventional surgery. One patient was transferred to a superior hospital to continue treatment.

### Outcomes

Among 940 patients, 182 (19.4%) patients died: 78 cases of respiratory failure (78/182, 42.9%), 34 of septic shock (34/182, 18.7%), 16 of heart failure (16/182, 8.8%), 18 of cerebrovascular disease (18/182, 9.9%), 10 of haemorrhagic shock (10/182, 5.5%), 6 of multiple organ failure (6/182, 3.3%), 5 of cardiopulmonary arrest (5/182, 2.7%), and 2 of hematological disease (2/182, 1.1%).

The prognosis patterns are shown in Table 2. The 28- (87.9% vs. 82.8%, *p* = .307) and 60-day (82.8% vs. 80.5%, *p* = .673) survival rates, ICU stay (12 vs. 9 days, *p* = .221), total hospital stay (24 vs. 17 days, *p* = .081), pulmonary embolism (none), and coagulopathy (5.2% vs. 4.4%, *p* > .999) were similar between the two groups; however, haemorrhage events were higher in the DVT group (37.9% vs. 6.7%, *p* < .001).

**Table 2.** Comparison of outcomes between DVT and non-DVT patients.

Prognosis	DVT group (*n* = 58)	Non-DVT group (*n* = 882)	*P*
28-day survival rate, *n* (%)	51 (87.9)	730 (82.8)	.307
60-day survival rate, *n* (%)	48 (82.8)	710 (80.5)	.673
ICU stay time, days, median (range)	12 (2–60)	9 (1–180)	.221
Total length of stay, days, median (range)	24 (3–120)	17 (2–180)	.081
Pulmonary embolism within 60 days	0	0	–
Haemorrhage event, *n* (%)	22 (37.9)	59 (6.7)	<.001
Coagulation dysfunction, *n* (%)	3 (5.2)	39 (4.4)	>.999

DVT: deep vein thrombosis; ICU: intensive care unit.

### Univariable and multivariable analysis of the risk factors for DVT

Before matching, the univariable analyses showed that chronic lung disease (*p* = .016), admission to a general ward before ICU (*p* = .005), admission to ICU due to internal medicine disease (*p* < .001), admission to ICU due to surgical medicine disease (*p* < .001), use of sedative and analgesic drugs (*p* = .006), use of muscle relaxants (*p* = .049), Caprini-high risk (*p* = .021), DVT assessment when the condition changed (*p* < .001), and physical prophylaxis (*p* < .001) were associated with DVT (Table 3).

**Table 3.** Univariable analysis of DVT.

Variables	Before matching			After matching		
	OR	95% CI	*P*	OR	95% CI	*P*
Chronic lung disease	1.176	1.042–1.173	.016	1.310	0.060–1.602	.162
Cerebrovascular disease	1.364	1.130–1.422	.055	1.284	1.086–1.941	.039
History of surgery	0.758	0.365–1.573	.457	1.324	1.133–1.788	.013
Admission to general ward before ICU	1.290	1.123–2.684	.005	0.841	0.264–2.673	.769
Admission to operating room before ICU	1.787	0.979–3.260	.059	3.302	1.187–9.180	.022
Admission to ICU due to internal medicine disease	1.374	1.216–1.647	<.001	1.752	0.358–1.578	.752
Admission to ICU due to surgical medicine disease	3.026	1.768–5.180	<.001	>0.999	0.481–2.079	>.999
Admission to ICU due to other reasons	1.360	1.360–1.366	.560	1.752	0.358–1.578	.752
Mechanical Ventilation	1.636	0.883–3.034	.118	2.065	0.929–4.596	.075
Sedative and analgesic drugs	2.444	1.300–4.598	.006	3.015	1.349–6.739	.007
Muscle relaxants	3.037	1.007–9.161	.049	1.735	0.041–5.641	>.999
APTT	1.002	0.991–1.014	.706	0.988	0.960–1.017	.422
INR	0.962	0.678–1.365	.827	0.901	0.503–1.612	.725
D–dimer	1.007	0.999–1.014	.081	1.998	1.132–1.990	.088
Caprini–high risk	1.966	1.110–3.482	.021	1.569	0.731–3.365	.248
DVT assessment within 24 h after admission	0.595	0.349–1.013	.056	0.611	0.292–1.280	.192
DVT assessment within 48 h after admission	0.871	0.337–2.254	.776	0.810	0.144–1.672	>.999
DVT assessment when condition changes	0.323	0.235–0.353	<.001	0.457	0.216–0.667	.001
Physiotherapy prophylaxis	0.822	0.370–0.819	.720	0.992	0.255–2.167	.558
Compression therapy	0.202	0.116–0.352	<.001	0.072	0.020–0.257	<.001

OR: odds ratio; CI: confidence interval; DVT: deep vein thrombosis; ICU: intensive care unit; APTT: activated partial thromboplastin time; INR: international normalised ratio.

After matching, cerebrovascular disease (*p* = .039), history of surgery (*p* = .013), admission to the operating room before ICU (*p* = .022), use of sedatives and analgesics (*p* = .007), DVT assessment when the condition changed (*p* = .001), and physical prophylaxis (*p* < .001) were associated with DVT (Table 3).

Before matching, the multivariable analysis showed that the D-dimer levels (OR = 1.011, 95% CI: 1.004–1.019, *p* = .003), DVT assessment when the condition changed (OR = 0.88, 95% CI: 0.42–0.63, *p* < .001), basic prophylaxis (OR = 0.126, 95% CI: 0.058–0.277, *p* < .001), and physical prophylaxis (OR = 0.156, 95% CI: 0.069–0.354, *p* < .001) were independently associated with DVT in ICU patients (Table 4).

**Table 4.** Multivariable analysis of DVT.

Variables	Before matching			After matching		
	OR	95% CI	*P*	OR	95% CI	*P*
Cerebrovascular disease	1.304	1.074–1.247	.098	1.069	1.004–1.086	.057
History of surgery	1.966	0.387–2.408	.940	1.973	0.231–4.106	.973
Admission to operating room before ICU	1.048	0.492–2.234	.904	1.588	0.291–8.661	.593
Mechanical Ventilation	1.383	0.427–4.479	.588	1.828	0.337–9.914	.484
Sedative and analgesic drugs	3.202	0.991–10.343	.052	0.979	0.170–5.632	.981
D–dimer	1.011	1.004–1.019	.003	1.256	1.132–1.990	.014
DVT assessment when condition changes	0.878	0.424–0.632	<.001	0.636	0.159–2.544	.532
Physiotherapy prophylaxis	0.126	0.058–0.277	<.001	0.092	0.016–0.536	.008
Compression therapy	0.156	0.069–0.354	<.001	0.159	0.038–0.674	.013

OR: odds ratio; CI: confidence interval; DVT: deep vein thrombosis; ICU: intensive care unit.

After matching, the multivariable analysis showed that the D-dimer levels (OR = 1.256, 95% CI: 1.132–1.990, *p* = .014), basic prophylaxis (OR = 0.092, 95% CI: 0.016–0.536, *p* = .008), and physical prophylaxis (OR = 0.159, 95% CI: 0.038–0.674, *p* = .013) were independently associated with DVT in ICU patients (Table 4).

## Discussion

DVT can lead to pulmonary embolism, and ICU patients are at high risk of DVT. The DVT prevention rate is one of the indicators of ICU care quality. However, currently, no specific assessment tool is available for evaluating the risk of DVT in general ICU patients. Moreover, domestic prevention of DVT lacks attention. Therefore, the lack of evaluation of DVT risk results in a low prevention rate. Furthermore, the epidemiology and the prevention and treatment patterns of DVT are poorly known in China. Therefore, this study aimed to investigate the prevention and treatment patterns of DVT in ICU patients in multiple hospitals of Zhejiang Province, China and to explore the risk factors for DVT in the Chinese population. The results revealed that the prevalence of DVT in ICU patients was 6.2%. The 28- and 60-day survival rates, ICU stay, total hospital stay, pulmonary embolism, and coagulopathy were similar between the two groups, but haemorrhage events were higher in the DVT group. The d-dimer levels, basic prophylaxis, and physical prophylaxis were independently associated with DVT in ICU patients.

Currently, Padua and Caprini risk scores are used as risk assessment tools for DVT in surgical patients. Therefore, it is necessary to understand the risk factors for DVT in general ICU patients and evaluate the existing scoring criteria of Padua and Caprini risk scores in such patients. the Caprini scale lacks some of the high-risk factors seen in ICU patients, such as infection, coma, and multiple organ dysfunction syndromes. In addition, some laboratory indicators that need gene testing that was included in the model are not routine inspection items in the ICU, resulting in the phenomenon of “no evaluation” or “default negative” results in their evaluation, which will affect the accuracy of the evaluation and may consequently underestimate the risk of ICU patients complicated with DVT. The Padua index is a simple indicator that is more suitable for medical patients and not for ICU patients. Currently, no study has shown the effectiveness of the Padua scale in evaluating DVT risk in ICU patients, and the Padua score is also insufficient. This score has only two tiers of risk stratification, which is not conducive for the management of DVT in ICU patients. The Wells DVT risk assessment form includes 10 items, including tumour, braking, surgery, DVT-related symptoms, and differential diagnosis. According to the total score, patients are divided into low risk (≤0 points), medium risk (1–2 points), and high risk (≥3 points). The Wells Score is used primarily to diagnose DVT in outpatients, and it is recommended to be combined with d-dimer. However, the items contained in the scale mostly reflect the existing symptoms and signs of DVT, and most ICU patients lack specific clinical manifestations. Whether the missed diagnosis rate of the Wells scale will affect the early prevention of ICU patients is also worth considering. In conclusion, due to the uniqueness of the ICU patients’ conditions and their clinical treatment, domestic and foreign guidelines only put forward that all critically ill patients at a high risk of DVT should receive prevention, but did not state any DVT evaluation criteria; therefore, no applicable specific screening scale exist for evaluating DVT in ICU patients.

In this study, the DVT prophylaxis rate was 90.1%. The most common prophylaxis was physical (75.0%), and 22.6% of patients received drug prophylaxis. According to the guidelines of the American College of Chest Physicians and the American Society of Haematology, thromboprophylaxis should be performed for patients in the ICU; physical prophylaxis is preferred for patients at higher risk of bleeding, while when the bleeding risk decreases, pharmacologic prophylaxis should be undertaken [25,26,31]. UFH, LMWH, and vitamin K antagonists are the most common drugs for prophylaxis. LMWH and UFH generally have a similar efficacy [12,32–35]. When pharmacological prophylaxis could be inadequate, mechanical prophylaxis, including the use of GCS and IPC devices, can be given [12,31]. Recent meta-analyses showed that mechanical prophylaxis is effective but there is a risk of pressure injury [33,36,37]. Nevertheless, a study in China showed that 25% of ICU medical staff never heard of mechanical DVT prophylaxis [38]. In addition, another review indicated that DVT prophylaxis is often overlooked [39]. The results of these studies highlight the need for training and education. In addition, the selection of the optimal prophylactic measure remains uncertain, especially in ICU patients with complex health conditions [12,40]. Nevertheless, the present study shows that both pharmacological and mechanical DVT prophylaxis was independently associated with lower ORs for DVT, indicating that they effectively reduce the DVT risk. This result is consistent with the findings of the study by Ejaz et al. [23] and guidelines of the American College of Chest Physicians and the American Society of Haematology [25,26,31]. Therefore, there is a need for proper training of the medical staff on the appropriate use of prophylactic measures to decrease the DVT risk. Nevertheless, in this study, the exact timing and the applied time of drugs or mechanical prophylaxis (i.e. mechanical prophylaxis first and then drugs) were not clearly stated.

Before matching, the variable of DVT assessment when the patient’s condition changed was associated with a lower DVT risk. Usually, a patient’s condition in the ICU is a dynamic process. All changes in the patient’s condition will change the various parameters that contribute to DVT risk, and intervening according to those changes should be conducive to effectively managing the risk of DVT, as supported by recommendations of the American Society of Haematology and the study by Adriance and Murphy [25,41]. Nevertheless, such an assessment was no longer associated with DVT after matching, which might be a statistical aberration. Therefore, this finding will have to be examined more closely in future research.

In the present study, only d-dimer levels were a risk factor for DVT. Indeed, research suggests that the measurement of d-dimer levels can be used to exclude the risk of VTE [42]. d-Dimer is the first-line test for screening patients at risk of DVT in emergency departments and ICUs. It is a fibrinolysis marker and has a high negative predictive value, making it a cost-effective triage method for DVT risk assessment [43,44]. Nevertheless, the cut-off value of d-dimer levels for the screening of DVT varies among patient populations (e.g. cancer patients, those in the second and third trimesters of pregnancy, and the elderly) [43,45], which might complicate its application in ICUs that receive a wide variety of patients. Therefore, future studies should examine d-dimer cut-off levels that could be effectively used in ICU patients.

Other work factors for DVT have been identified in the literature, but not in this study. In a neurologic ICU, paralysis and pulmonary infection were the main risk factors for DVT [46]. In a few studies, the Caprini risk score was shown to be a good predictor of VTE [27,47], but it was validated in general surgery patients and might not be appropriate for ICU patients, possibly explaining why it was not identified as a risk factor in this study. Generally accepted risk factors for DVT include cancer, surgery, age, immobilisation, obesity, history of VTE, sepsis, stroke, lung failure, heart failure, pregnancy, and trauma [5–10,31,48–51]. However, these factors were not observed to be associated with DVT in the present study probably because this study included a general ICU population with various conditions, diluting the impact of risk factors specific to a given condition. Considering the differences in patient care required in ICUs, the physical prevention of DVT needs the joint efforts of respiratory, nursing, and rehabilitation staff.

This study has several strengths. First, this study analyzes data from China, providing valuable information about the prevention, treatment, and risk factors of DVT in China. Second, this is a multi-center study, including a varied population from Zhejiang Province. Finally, the study includes a large sample size, providing significant statistical power. This study also has some limitations. As an observational study, the definite preventive effects of DVT prophylaxis could not be determined, and randomised clinical trials are needed to provide high-level evidence for further analysis. Second, this study was conducted in the ICUs in Zhejiang Province only; therefore, the geographical scope of this study is not wide enough to allow generalisability to the entire country. Therefore, replicating this study across a larger patient population would ensure the broader applicability of the results. Third, the VTE outcomes included the length of hospitalisation, leading to underestimating the VTE risk in this patient population. This study observed only patients with VTE admitted to the ICU and did not evaluate them during follow-up visits after leaving the ICU. In future studies, the development of ICU-specific DVT assessment tools or models using big data analytics or artificial intelligence and the addition of risk stratification management to refine prevention and treatment methodologies should be considered.

## Conclusions

In conclusion, DVT prophylaxis is widely performed in ICU patients, except for those at high risk of bleeding. Prophylaxis is an independent protective factor for DVT occurrence. The most common treatment of DVT patients is LMWH, although it might increase the rate of bleeding. The short-term survival is similar between DVT and non-DVT patients. Further multicenter, prospective, non-randomised controlled studies are needed to develop a standardised training of prophylaxis measures for DVT.

## Supplementary Material

Supplemental Material

## Data Availability

The authors confirm that the data supporting the findings of this study are available within the article.
