# Supplementary material for: Prevention, treatment, and risk factors of deep vein thrombosis in critically ill patients in Zhejiang province, China: a multicenter, prospective, observational study
Source: Ann Med. 2021 Nov 19;53(1):2236–47. doi: 10.1080/07853890.2021.2005822 (PMC8805816; doi:10.1080/07853890.2021.2005822)
Supplement: Supplemental Material [file IANN_A_2005822_SM4925.zip › Supplemental files/Supplementary Table S1.docx]

**Supplementary Table S1.** Characteristics of the 54 participating hospitals

| **Hospital characteristics** | **Value** |
| --- | --- |
| Hospital level, n (%)  Grade-3  Grade-2 | 35 (64.8%)  19 (35.2%) |
| Number of beds in ICU, mean ± SD | 24.5±11.1 |
| ICU doctor bed ratio, mean ± SD:1 | 0.53±0.18:1 |
| ICU nurse bed ratio, mean ± SD:1 | 1.98±0.49:1 |
| Average number of ICU patients admitted per year, mean ± SD | 5282.2±3044.5 |
| ICU admission ratio | 0.02±0.01 |

ICU doctor bed ratio: the ratio of the number of ICU doctors to the number of ICU beds; the ratio of ICU nurse beds: the ratio of the number of ICU nurses to the number of ICU beds; ICU admission ratio: the ratio of the number of ICU admission to the number of total hospital admission.
