# Supplementary material for: Prevention, treatment, and risk factors of deep vein thrombosis in critically ill patients in Zhejiang province, China: a multicenter, prospective, observational study
Source: Ann Med. 2021 Nov 19;53(1):2236–47. doi: 10.1080/07853890.2021.2005822 (PMC8805816; doi:10.1080/07853890.2021.2005822)
Supplement: Supplemental Material [file IANN_A_2005822_SM4925.zip › Supplemental files/Supplementary Table S2.docx]

**Supplementary Table S2.** Participating hospitals and physicians

| **Hospital** | **Physicians** |
| --- | --- |
| Department of Critical Care Medicine, Xinchang Hospital of Traditional Chinese Medicine | Bichun Lv |
| Department of Critical Care Medicine, Dongyang People’s Hospital | Yongxia Hu |
| Department of Critical Care Medicine, The Second Affiliated Hospital Zhejiang University School of Medicine | Man Huang, Chengyang Chen |
| Department of Critical Care Medicine, Ninghai First Hospital | Kangmin Xu |
| Department of Critical Care Medicine, The Second Affiliated Hospital and Yuying Children’s Hospital of WMU | Yu Hao |
| Department of Critical Medicine, Huzhou Central Hospital, Affiliated Hospital of Huzhou Normarl University | Xiaowei Ji, Yongbin Wang, Jianhong Lu |
| Department of Critical Medicine, Anji People’s Hospital | Zhongxiang Yao, Nianbin Ma |
| Department of Critical Medicine, Zhoushan Hospital | Keqi Dong, Miao Liu |
| Department of Critical Medicine, Yuhang Hospital of TCM | Xiqing Zuo, Liqun Cui |
| Department of Critical Medicine, Zhejiang Hospital | Caibao Hu |
| Department of Critical Medicine, The First People’s Hospital of Huzhou | Kankai Tang, Zhidong Chen |
| Department of Critical Medicine, Zhejiang Quhua Hospital | Yuezhen Liu, Binbin Wu |
| Department of Critical Medicine, Jinhua Central Hospital | Wei Peng |
| Department of Critical Medicine, Quzhou People’s Hospital | Danqiong Wang |
| Department of Critical Medicine, Changshan People’s Hospital | Youqin Yan |
| Department of Critical Medicine, The First People’s Hospital of Xiaoshan | Xiaoyuan Shen, Jiawei Lai |
| Department of Critical Medicine, Xianju People’s Hoaspital | Xiaoming Zhang, Mengting Li |
| Department of Critical Medicine, The Second Affiliated Hospital of Zhejiang Chinese Medical University | Jun Lu, Zhaoqing Jiang |
| Department of Critical Medicine, The first Hospital of Jiaxing | Jiangang Zhu, Peng Shen, Wenlong Wei |
| Department of Critical Medicine, The First People’s Hospital of Fuyang | Weidong Tang, Yanan Zhang |
| Department of Critical Medicine, The Second People’s Hospital of Yuhuan | Weimin Lin, Xinfang Xie |
| Department of Critical Medicine, Kaihua Hospital of TCM | Shengwu Fu, Han Xue |
| Department of Critical Medicine, The Affiliated Hospital of Hangzhou Normal University | Leqing Lin, Liang Guo |
| Department of Critcal Medicine, Taizhou Central Hospital | Yuhang Lv, Danfeng Weng |
| Department of Critical Medicine, Tongde Hospital of Zhejiang Province | Zhizhen Lai, Linxiang Sheng |
| Department of Critical Medicine, Yiwu Central Hospital | Mingxia Ji, Jiao Wang, Lidan Chen |
| Department of Critical Medicine, Zhejiang Provincial Hospital of Chinese Medicine | Zhirong Zhang, Zhaokun Fan |
| Department of Critical Medicine, Zhejiang Provincial People’s Hospital | Minhua Chen, Ziqiang Shao |
| Department of Critical Medicine, Zhejiang Provincial Hospital of Chinese Medicine | Dandan Feng |
| Department of Critical Medicine, Affiliated Hangzhou First People’s Hospital, Zhejiang University School of Medicine | Ying Zhu, Mingyue Chen |
| Department of Critical Medicine, Ningbo First Hospital | Zhiyu Wang, Yangyang Ying |
| Department of Critical Medicine, Taizhou Hospital of Zhejiang | Sheng Zhang, Weixing Yang |
| Department of Emergence Critical Medicine, The Second Hospital of Jiaxing | Tao He, Li Sun, Lu Bai |
| Department of Critical Medicine, The Second People’s Hospital of Yuhang | Haiyan Lu, Dinghao Weng |
| Department of Critical Medicine, Yuyao People’s Hospital of Zhejiang Province | Ting Shen, Yabo Huang |
| Department of Critical Medicine, Shaoxing People’s Hospital | Guofeng Yu, Zhixin Li |
| Department of Critical Medicine, Affiliated Hangzhou First People’s Hospital, Zhejiang University School of Medicine | Yongke Zheng, Kai Qiu |
| Department of Critical Medicine, The First People’s Hospital of Yuhang | Yunlong Wu, Jin Zhou |
| Department of Critical Medicine, Xinchang People’s Hospital | Ziqiang Ming, Peifeng Qiu |
| Department of Critical Medicine, Kecheng People’s Hospital | Wei Chen, Qinglong Feng |
| Department of Critical Medicine, Lishui People’s Hospital | Yiping Ning, Ke Li |
| Department of Critical Medicine, Xixi Hospital of Hzngzhou | Siquan Zhang, Xudong Le |
| Department of Critical Medicine, Lvcheng Hospital | Buqing Ma, Long Chi |
| Department of Critical Medicine, Jingning People’s Hospital | Zhengchun Wu, Han Wu |
| Department of Critical Medicine, The Red Cross Hospital of Hangzhou | Zhihui Li, Zheng Yang |
| Department of Critical Medicine, Hangzhou of Traditional Chinese Medicine | Meiqi Zhang, Weihua Wu |
| Department of Critical Medicine, The First People’s Hospital of Tonglu | Xiaoping Wu, Xin Jiang |
| Department of Critical Medicine, Cangnan People’s Hospital | Yan Liang, Xianyuan Chen |
| Department of Critical Medicine, Longyou People’s Hospital | Yandong Cheng, Jun Lu |
| Department of Critical Medicine, Pujiang People’s Hospital | Fengyang Yang, Junjie Xie |
